# Functional (Psychogenic) Movement Disorders Presenting During Sleep

**DOI:** 10.5334/tohm.571

**Published:** 2021-02-22

**Authors:** José Fidel Baizabal-Carvallo, Marlene Alonso-Juarez, Robert Fekete

**Affiliations:** 1 Department of Sciences and Engineering, University of Guanajuato, México; 2 National Polytechnic Institute, México; 3 New York Medical College, Valhalla, New York, USA

**Keywords:** Functional movement disorder, dystonia, sleep, polysomnography, trauma-associated sleep disorder

## Abstract

**Background:**

Functional (psychogenic) movement disorders are involuntary movements that seems to originate from activation of voluntary motor pathways in the brain. The movements typically present during the waking hours with variable frequency.

**Case presentation:**

We present the case of a 24-year-old woman with FMDs during the waking state, but also during stages 1 and 2 of non-REM sleep and REM sleep, recorded with polysomnography. Such movements caused arousal leading to excessive daytime sleepiness and fatigue.

**Conclusions:**

FMDs may disrupt sleep causing day time somnolence, adding morbidity to the disorder.

## Introduction

Functional (previously known as psychogenic) movement disorders (FMDs) are involuntary movements modifiable by attention and distraction that may present with variable clinical phenomenology. Several lines of evidence suggest that FMDs originate from activation of voluntary motor pathways; however, it is unclear why they are perceived by patients as involuntary [1]. Several psychodynamic theories have been proposed to explain the pathogenesis of FMDs; however, these theories have not been able to provide a full explanation for the development of these movements and modern functional neuroimaging studies suggest that patients suffering FMDs have a distinct abnormal activation of cortical and subcortical brain structures compared to normal controls [2,3]. It is unclear whether this anomalous activation predispose to abnormal movements or results from them. Moreover, it is uncertain whether such abnormal brain activation may present, in some cases, during states of decreased physiological consciousness such as sleep, leading to the appearance of FMDs. Here, we report a patient with documented FMDs during sleep and discuss possible underlying pathogenesis.

## Case Report

A 24-year-old, right handed, Hispanic woman, presented for evaluation of a 6-month history of episodic axial and appendicular dystonia, causing severe opisthotonus and twisted postures in the upper and lower limbs of variable duration lasting from few seconds to 3–4 hours, the episodes were sometimes preceded by stereotypic finger movements and sometimes accompanied by hand tremor and severe stuttering. These episodes started suddenly after a minor car accident and presented several times a day. The presence of marked suggestibility, distractibility, prominent stuttering associated with the dystonic episodes [4]; sudden onset of abnormal movements with periods of unexplained improvement; lack of family history of dystonia and normal brain magnetic resonance imaging were consistent with FMDs. No abnormalities in cranial nerves, muscle strength, deep tendon reflexes, sensation and gait were documented in this patient. She underwent a video-EEG (VEEG) examination awake (6 h) showing multiple spells of abnormal dystonic movements with lack of electroencephalographic correlation.

The patient’s parents noted similar dystonic movements while the patient was sleeping almost every night. They denied similar movements during sleep before the onset of FMDs. There was no personal or family history of parasomnias such as sleepwalking, night terrors, or confusional arousals. The patient complained of fragmented sleep, as the movements wake her up 2 to 3 times per night causing fatigue and excessive daytime sleepiness. She scored 20 out of 24 points in the Epworth Sleepiness Scale (ESS). There were no complains of snoring, nocturia or myoclonic movements during the sleep time. The patient denied drinking coffee, tea or soft drinks before going to sleep. Sleep habits included a consistent bedtime at 11:30 p.m. and awakening at 7.30 a.m., daily naps of 2 hours during the afternoon were also endorsed by the patient.

**Figure 1:**
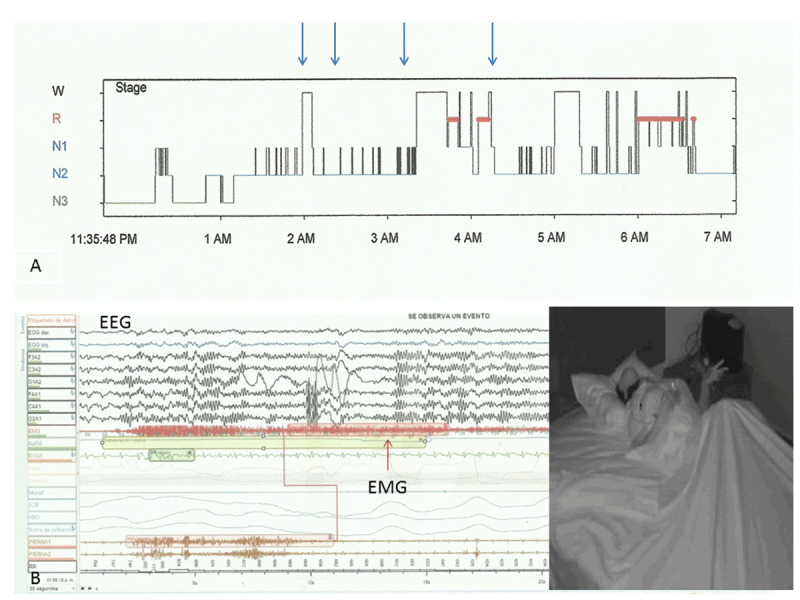
**A)** Hypnogram for PSG-1 showing the occurrence of dystonic episodes (arrows). NREM: E1: stage 1, E2: stage 2, E3: stage 3, R: REM; W: awake. **B)** Episode of dystonia which translates into EMG activity, occurring during the NREM-E2; EEG shows multiple sleep spindles and K complexes consistent with this phase of sleep; picture shows axial and upper limb dystonia registered in the EMG.

The patient underwent 2 overnight polysomnographs (PSG) (Alice 5 type 1, Respironics Inc., Murrysville, Penn. USA), according to the guidelines of the American Academy of Sleep Medicine (www.aasmnet.org). In the first PSG study, there was a delayed rapid eye movement (REM) sleep, with a latency of 248 minutes; there were 2 episodes of mild distal upper and lower limb spasms during non-REM sleep stage 2, lasting 37 and 40.5 seconds respectively (***Figure 1***), the first episode was related to awakening, in the second episode the patient remained in stage 1; a third episode with similar features presented during non-REM sleep stage 1 lasting 18 seconds. The patient remained in stage 1 after the movements subsided. A fourth episode of upper limb and axial dystonia lasting 30 seconds was observed during REM sleep, followed by awakening (***Figure 1***). There were no other sleep-related breathing or movement disorders (***Table 1***). In a second PSG carried out two weeks later, two episodes of mild distal dystonic movements presented during non-REM sleep stage 1 followed by arousal and severe generalized dystonia (see ***video***). All episodes observed during PSG were clinically similar to those observed during video-EEG and none of them was related to electroencephalographic evidence of epileptic activity.

**Table 1:** Summary of sleep parameters during the PSG-1.


	TIME AND NUMBER

Total sleep time (TST= REM+NREM)	399 min

Sleep efficiency: Time asleep/(total time in bed-time to fall sleep)	87.7%

Total number of arousals	95

Arousal index	14.3/h

Distribution of sleep stages	

Awake	56 min

NREM Total	357 min

E1	57 min

E2	231 min

E3	69 min

E4	0 min

REM	42 min

Sleep latencies	

E1	37.5 min

REM	248 min

Periodic limb movement during sleep	0

Total number of lower limb movements	24

Snoring	

Total number of snoring episodes	9

Mean duration of snoring episodes	3.4 sec

Total time snoring	0.5 min

Apnea/hypopnea Index (Apnea:hypopnea/TST)	

NREM	0.2/h

REM	0.0/h

TST	0.2/h


REM: rapid-eye movements; non-REM sleep stages: E1: stage 1, E2: stage 2, E3: stage 3, E4: stage 4. TST: total sleep times.

**Video:** Patient shows hand stereotypes and dystonia followed by axial dystonia with opisthotonus during early phases of non-REM sleep. These episodes were registered by EMG as shown in figure 1B.

An initial trial of high doses of antiepileptic drugs was provided elsewhere including levetiracetam 1500 mg twice a day, phenytoin 100 mg three times a day and valproate 600 mg three times a day for two weeks each, without any benefit and they were discontinued. At the initial evaluation by us, the patient was receiving treatment with escitalopram oxalate 20 mg per day without any benefit in her abnormal movements.

However, treatment with physiotherapy, psychotherapy plus alprazolam 1 mg per night then substituted by clonazepam up-to 4 mg per night and mirtazapine from 7.5 to 22.5 mg per night, provided marked improvement with disappearance of sleep fragmentation, dystonic movements during sleep and excessive daytime sleepiness within the first 3 months after starting multidisciplinary treatment, whereas movements awake had a slower but progressive improvement within the next 10 months. The patient has been without mirtazapine and benzodiazepines within the last 5 to 6 months without recurrence of sleep and awake movements.

## Discussion

We report a patient fulfilling diagnostic criteria for “documented” and “clinically established” FMDs according to the Fahn and Williams criteria [5]. She showed similar dystonic movements during early phases of non-REM sleep and REM-sleep, disrupting her sleep cycles leading to daytime sleepiness and fatigue. After extensive investigations with VEEG and PSG, there was no evidence of some forms of epilepsy including frontal lobe epilepsy which it may present with seizures during sleep. The spells during sleep improved without anti-epileptics, and improvement persisted after discontinuing benzodiazepines and mirtazapine. Moreover, there was no evidence either of epileptic seizures while awake. Some paroxysmal movement disorders such as those related to MR1, KCNMA1 and ATP1A3 mutations may improve with benzodiazepines; however, these disorders show a much younger age at onset (around 5 years), have clear precipitants, do not seem to present during sleep and they should recur following discontinuation of benzodiazepines.

A number of sleep-related movement disorders have been recognized according to the *International Classification of Sleep Disorders* third edition (ICSD3) [6], including: restless legs syndrome, sleep-related leg cramps, sleep bruxism, sleep-related leg cramps, benign sleep myoclonus of infancy, periodic limb movement disorder, sleep-related rhythmic movement disorders, and propriospinal myoclonus (PSM); however, the majority of these movements have a myoclonic character and a rhythmic or periodical presentation instead of the dystonic and non—rhythmic pattern registered in our patient [7]. The ICSD3 also categorizes “sleep-related movement disorders” as “unspecified”, they may be due to medication or medical disorder; we consider that our patient fits into the latter category. Trauma associated sleep disorder (TASD) is a recently described condition characterized by: previous traumatic experience, nightmares related to the traumatic event, dream enactment behaviors during REM and non-REM sleep, autonomic hyperarousal and lack of epileptic activity on EEG [8,9]. Some of these features were observed in our patient with the notable lack of nightmares related to the traumatic event, whether our patient’s manifestations are a subtype of TASD or a different disorder is uncertain.

PSM is a movement disorder occurring during the sleep-wake transition [10]. Although PSM was considered a variant of organic axial myoclonus, a substantial proportion of patients with PSM are believed to have a functional neurological disorder, based on the observation that the movements can be imitated voluntarily and the presence of *Bereitschaftspotential* preceding PSM [11]. For this reason, PSM may be an example of a FMD presenting in the sleep-wake transition that can potentially disrupt sleep.

On the other hand, sleep-related bruxism consists in rhythmic contractions of the masticatory muscles leading to tooth grinding, typically occur in light non-REM sleep and during REM sleep [7]; evidence suggests that these episodes are related to micro-arousal [12]. Both features were also observed in our patient, but with movements similar to those experienced while awake during an attack of functional dystonia and stereotypies. Interestingly, our patient had dystonic movements during REM sleep with lack of atonia, we were not able to confirm dream enactment; however, these features suggest the presence REM sleep behavior disorder (RBD); although this disorder frequently heralds the onset of a neurodegenerative disorders, it may occur associated with certain medications such as selective serotonin reuptake inhibitors (SSRIs), or neurological disorders including narcolepsy, Tourette syndrome (TS) [6] and possibly FMDs as demonstrated by this case.

Movements during sleep, similar to those observed during awakening in patients with FMDs may be related to a state of hyperarousal and decreased intracortical inhibition involving motor pathways; both abnormalities have been documented in patients with FMDs [13,14]; and have also been proposed to explain the increased motor activity and tics during REM and non-REM sleep observed in patients with TS [15]. Current evidence suggests that patients with FMDs may have abnormally enhanced connections between limbic/emotional and motor systems [16]; making the latter more prone to activate under certain external or internal stimuli; this activation may possibly occur during different sleep stages or during the sleep-wake transition.

In our patient, improvement was observed following a multidisciplinary therapy with dystonic movements during sleep disappearing before FMDs while awake. Suggesting that movements during sleep are more susceptible to disappear with appropriate treatment; however, deepening of sleep induced by benzodiazepines may have also contributed to such improvement.

In conclusion, abnormal movements disrupting sleep or sleep-awake transition causing daytime sleepiness may be present in some patients with FMDs. Besides their impact in sleep quality, they may lead to microarousals, daytime sleepiness and fatigue [17]. The presence of such movements during sleep, challenges the notion that these movements would only present during the waking state. The patient also showed dystonic movements during REM sleep, suggesting the presence of RBD, possibly expanding the list of disorders related to this parasomnia and overlapping with TASD. Further studies should help to determine how frequently these phenomena occur during sleep and their clinical correlations in patients with FMDs. We are not aware of previous reports of FMDs during sleep; we encourage clinicians and researchers to look for signs of disrupted sleep and document abnormal movements during sleep in these patients.

## Data Accessibility Statements

Data is available on request to the authors.

## Abbreviations

EEG: electroencephalogram

FMDs: Functional movement disorders

ESS: Epworth Sleepiness Scale

PSM: propriospinal myoclonus

REM: rapid eye movement

PSG: polysomnographs
